# Circular RNA VANGL1 knockdown suppressed viability, promoted apoptosis, and increased doxorubicin sensitivity through targeting miR-145-5p to regulate SOX4 in bladder cancer cells

**DOI:** 10.1515/med-2021-0299

**Published:** 2021-07-06

**Authors:** Jiangbo Zhu, Fei Zhang

**Affiliations:** Department of Urology, Taizhou First People’s Hospital, Huangyan District, 318020, Taizhou, China

**Keywords:** circ_VANGL1, miR-145-5p, SOX4, doxorubicin, bladder cancer

## Abstract

**Background:**

Bladder cancer is a common malignancy in the world. It is reported that circular RNA VANGL1 (circ_VANGL1) was involved in bladder cancer progression. However, the functional role and molecular mechanism of circ_VANGL1 in bladder cancer were still unclear.

**Methods:**

The levels of circ_VANGL1, microRNA-145-5p (miR-145-5p), and Sex-determining region Y-related high-mobility group box 4 (SOX4) in bladder cancer tissues and cells were determined by quantitative real-time polymerase chain (RT-qPCR). The relative protein expression was detected by western blot. Cell counting kit-8 (CCK8) and flow cytometry analysis were used to measure cell viability, IC_50_ value, and apoptosis rate. The interaction between miR-145-5p and circ_VANGL1 or SOX4 was predicted by online software starBase v2.0 or Targetscan and verified by the dual-luciferase reporter assay. Besides, xenograft mice model was used to detect the effects of circ_VANGL1 *in vivo*.

**Results:**

The level of circ_VANGL1 and SOX4 was increased, while miR-145-5p was decreased in bladder cancer tissues and cells. Knockdown of circ_VANGL1 suppressed viability, while promoted apoptosis and increased doxorubicin sensitivity in bladder cancer cells. Moreover, circ_VANGL1 acted as a sponge for miR-145-5p. In addition, miR-145-5p partially reversed the effects of miR-145-5p knockdown in T24 and J82 cells. SOX4 was a target of miR-145-5p and negatively regulated by miR-145-5p. Furthermore, miR-145-5p regulated SOX4 to affect cell progression in bladder cancer cells, including viability, apoptosis, and doxorubicin sensitivity. Besides, circ_VANGL1 suppressed tumor growth and enhanced the doxorubicin sensitivity in bladder cancer *in vivo*.

**Conclusion:**

circ_VANGL1 mediated cell viability, apoptosis, and doxorubicin sensitivity by regulating miR-145-5p/SOX4 axis in bladder cancer, providing a potential therapeutic target for bladder cancer therapy.

## 1 Introduction

It was reported that there were an estimated 74,690 new cases and 15,580 deaths due to bladder cancer in 2014 in USA [1]. Many factors contributed to bladder cancer, such as increasing age, smoking, or some industrial chemicals [2]. Although surgical resection and chemotherapy are the main therapies for bladder cancer patients, drug-resistance contributes to a critical barrier in bladder cancer therapy. Doxorubicin (DOX) is a natural anthracycline antibiotic and widely used in systemic chemotherapy and intravesical for bladder cancer. Therefore, the molecular mechanisms that regulate bladder cancer development and chemosensitivity are essential for further study.

Emerging evidence indicates that circRNAs are involved in the progression of many diseases, including cancers. A previous research indicated that circRIP2 exerted promotion effects on epithelial-mesenchymal transition in bladder cancer cells [3]. Moreover, circRNA ITCH suppressed bladder cancer development through regulating the expression of p21 and PTEN by targeting miR-224/miR-17 [4]. High-throughput microarray analysis and previous evidence suggested that circRNA VANGL1 (circ_VANGL1) (circBase ID: hsa_circ_0002623) was aberrantly expressed in bladder cancer. However, the functional role and the underlying mechanism in bladder cancer remain unknown.

circRNAs can act as microRNAs (miRNAs) sponges to regulate gene transcription and translation by inhibiting the activity of miRNA [5]. miRNAs, with 19-22 nucleotide, are noncoding RNAs that decrease translation of proteins or promote degradation of the mRNA by targeting 3′-untranslated region (3′-UTR) of mRNA [6]. It is reported that an estimated 60% of the mRNA have potential conserved sites that interact with miRNAs [7]. Nowadays, approximately 2,000 human miRNAs were identified and recorded by the miRBase databases [8]. Present evidence demonstrated that miRNAs play vital roles in various biological processes, including cell proliferation, apoptosis, and migration in human cancers [8,9]. microRNA-145-5p (miR-145-5p), a endogenous miRNA, played multiple roles in various human cancers, such as colorectal cancer [10], breast cancer [11], gastric cancer [12], and bladder cancer [13]. However, whether miR-145-5p participated in the regulatory mechanism of circ_VANGL1 in bladder cancer is still unclear.

In our present research, the expression of circ_VANGL1 in bladder cancer tissues and cells was detected. Moreover, we explored the functional role of circ_VANGL1 and its potential regulatory mechanism in the progression of bladder cancer.

## 2 Materials and methods

### 2.1 Tissue samples and cell culture

A total of 36 bladder cancer tissues and the matched adjacent normal tissues were isolated from the bladder cancer patients who underwent surgery at Taizhou First People’s Hospital. The study was approved by the Ethics Committee of Taizhou First People’s Hospital.

Two human bladder cancer cell lines (T24 and J82) and normal cell line (SV-HUC-1) were purchased from American Tissue Culture Collection (ATCC, Manassas, VA, USA). All cells were cultured in Dulbecco’s Modified Eagle Medium (DMEM; Invitrogen, Carlsbad, CA, USA) containing 10% fetal bovine serum (FBS; Thermo Fisher Scientific, Waltham, MA, USA), incubated in an incubator with 95% air and 5% CO_2_ at 37°C.

### 2.2 Plasmid construction and cell transfection

The sequence of Sex-determining region Y-related high-mobility group box 4 (SOX4) was cloned into pcDNA3.1 (Invitrogen) to construct SOX4 overexpression vector, termed pcDNA3.1-SOX4 (pc-SOX4). 2 × 10^5^ T24 and J82 cells were added into a 12-well plate. Then, 0.2 μg of pc-SOX4 or pc-NC was transfected into T24 and J82 cells using 0.5 μL of Lipofectamine 3000 reagent (Invitrogen, Carlsbad, CA, US). Small interference RNA against circ_VANGL1 (si-circ) and its control (si-NC), miR-145-5p inhibitor and the negative control (NC inhibitor), miR-145-5p mimic, and the blank control (NC-mimic) were purchased from RIBOBIO (Guangzhou, China). These aforementioned oligonucleotides (0.5 μg) were transfected into T24 and J82 cells with 0.6 μL of Lipofectamine 3000 (Invitrogen).

### 2.3 RNA extraction and quantitative reverse transcription-PCR (RT-qPCR)

Total RNA was isolated from tissues or cells using TRIzol (Invitrogen, Carlsbad, CA, USA) according to the manufacturer’s instructions. RNA concentration was verified before the reverse transcription reaction that was carried out using Prime Script RT reagent Kit (Takara, Dalian, China). Quantitative real-time polymerase chain reaction (RT-qPCR) was performed with SYBR green (Applied Biosystems, Foster City, CA, USA). The relative expression of genes was analyzed using U6 or GAPDH as reference gene by 2^−ΔΔCt^ method. The primes for circ_VANGL1, miR-145-5p, SOX4, U6, and GAPDH were listed as follows: circ_VANGL1 (forward primer: 5′-GTCCGCTCCACCGATGGCGA-3′, reverse primer: 5′-CTGAACTTCCTCTGTCCGAGT-3′), miR-145-5p (forward primer: 5′-CAGTCTTGTCCAGTTTTCCCAG-3′, reverse primer: 5′-TATGCTTGTTCTCGTCTCTGTGTC-3′), SOX4 (forward primer: 5′-GGCCTGTTTCGCTGTCGGGT-3′, reverse primer: 5′-GCCTGCATGCAACAGACTGGC-3′), U6 (forward primer: 5′-CTCGCTTCGGCAGCACA-3′, reverse primer: 5′-AACGCTTCACGAATTTGCGT-3′), GAPDH (forward primer: 5′-CCGGGAAACTGTGGCGTGATGG-3′, reverse primer: 5′-AGGTGGAGGAGTGGGTGTCGCTGTT-3′).

### 2.4 Cell viability and apoptosis assay

The Cell Counting Kit-8 (CCK-8; Dojindo Molecular Technologies, Kumamoto, Japan) was used to detect cell viability. 2 × 10^4^ T24 and J82 cells were digested and cultured in a 96-well plate. After transfection, the cells were incubated for 48 h and then the WST-8 reagent was added with a final concentration of 0.5 mg/mL. After another 2 h of incubation at 37°C, cell absorbance was measured at 450 nm using a microplate reader (Bio-Rad, Richmond, CA, USA).

Cell apoptosis assay was performed using the Annexin V-Fluorescein (FITC) Apoptosis Detection Kit (Solarbio, Beijing, China). First, cells were collected and washed by 1× Binding Buffer for 2 times and then were resuspended using 1× Binding Buffer with a final concentration of 1 × 10^6^ cells/mL. Next, 5 µL of Annexin V-FITC and 5 µL of propidium iodide (PI) solution were added to 100 µL cell suspension and cultured for 15 min at room temperature in the dark. Finally, the samples were detected by a FACScan flow cytometry (BD Biosciences, San Jose, CA, USA).

### 2.5 Chemotherapy resistance assay

T24 and J82 cells were treated with doxorubicin at various concentrations (1, 0.1, 0.01, 0.001, and 0.0001 ng/mL) for 24 h. The cell viability was determined by CCK-8 assay 48 h later. Based on the data, the dose-response curve was charted and the half maximal inhibitory concentration (IC_50_) was determined using SPSS software.

### 2.6 Western blot assay

Total proteins were extracted using RIPA buffer (Beyotime, Shanghai, China) and separated by dodecyl sulfate, sodium salt-polyacrylamide gel electrophoresis (SDS-PAGE, 10%), then electrotransferred onto a polyvinylidene didluoride (PVDF) membrane (Millipore, Billerica, MA, USA). The membrane was blocked in 5% skimmed milk at room temperature for 1 h and then incubated with primary antibody at 4°C overnight. After washing with TBST (Tris-buffered saline containing 0.1% Tween-20) buffer for three times, the membrane was incubated with horseradish peroxidase-conjugated secondary antibody at room temperature for 1 h. The chemiluminescent immunoassay was used to detect protein signaling. Primary antibodies used in this study, including c-caspase-3 (1:1,000, ab2302), ki-67 (1:5,000, ab92742), SOX4 (1:1,000, ab90696), and GAPDH (1:1,000, ab181602), were purchased from Abcam (Cambridge, UK).

### 2.7 The dual-luciferase reporter assay

The potential binding sites between miR-145-5p and circ_VANGL1 or SOX4 were predicted by online software starBase v2.0 (http://starbase.sysu.edu.cn/starbase2/) or TargetScan (http://www.targetscan.org/vert_72/). The wild-type circ_VANGL1 or SOX4-3′UTR sequence containing the putative binding sites and mutated circ_VANGL1 or SOX-3′UTR sequence were amplified through PCR and cloned into pGL3 vector (Promega, Madison, WI, USA), namely circ_VANGL1 wt, circ_VANGL1 mut, SOX4 3′UTR wt, or SOX4 3′UTR mut. Next, the reporter vectors and miR-145-5p mimic or NC-mimic were co-transfected into T24 cells and J82 cells. After incubation for 48 h, the luciferase activity was determined by the Lmax multiwall luminometer (Molecular Devices, LLC, Sunnyvale, CA, USA), followed by normalizing with Renilla luciferase activity.

### 2.8 Nude mice experiments

All the experiments were approved by the Animal Care and Use Committee of Taizhou First People’s Hospital and performed following guidelines of the national animal protection and ethics institute. Twenty BALB/c nude mice (4 weeks old) bought from Vital River Laboratory Animal Technology (Beijing, China) were used in this study. All the mice were housed in SPF animal room and water, food, and padding were regularly replaced. T24 cells or J82 cells were transfected with short hairpin against circ_VANGL1 (sh-circ) to establish the stable T24 or J82 cell line. 5 × 10^6^ T24 cells, J82 cells, or stable T24 or J82 cells were subcutaneously injected to the forelimb axils of nude mice. The nude mice were divided into 4 groups (6 mice per group): Control, Dox, sh-circ, or sh-circ + Dox. The mice in Dox and sh-circ + Dox group were injected with 3 mg/kg Dox twice per week, while the mice in Control and sh-circ were treated with the equal amount of saline. Tumor volume was measured every 3 days. After 15 days, the mice were euthanized, and tumor tissues were weighted and stored for further research. Tumor volume was computed by the equation *V* (mm^3^) = (width)^2^ × length/2.

### 2.9 Immunohistochemistry analysis

Immunohistochemistry was performed using antibodies against ki-67 (1:500, ab92742, Abcam). Tissue samples of 5 μm thick paraffin section were stained with IHC. The immunostaining images were captured using Olympus FSX100 microscope (Olympus, Japan).

### 2.10 Statistical analysis

All data were expressed as mean ± standard deviation of at least three independent experiments. Statistical analyses were performed by SPSS 22.0 software. Student’s *t*-test or one-way analysis of variance (ANOVA) was utilized to evaluate the difference between two groups or among multiple groups, respectively. *P* < 0.05 was considered to be statistically significant.

## 3 Results

### 3.1 circ_VANGL1 was increased in bladder cancer tissues and cells

**Figure 1:**
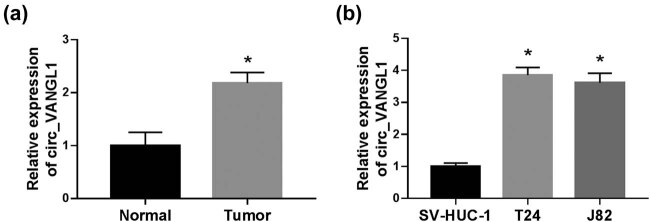
The level of circ_VANGL1 in bladder cancer tissues and cells. (a) RT-qPCR was used to detect the level of circ_VANGL1 in tumor tissues (*n* = 36) and matched normal tissues (*n* = 36). (b) The level of circ_VANGL1 in bladder cancer cells (T24 and J82) and human normal bladder epithelial cell line (SV-HUC-1) was measured by RT-qPCR. *P < 0.05.

To explore the role of circ_VANGL1 in bladder cancer, we detected the expression of circ_VANGL1 in bladder cancer tissues and cells. The result showed that circ_VANGL1 was highly expressed in tumor tissues (*n* = 36) compared with normal tissues (*n* = 36) (Figure 1a). Consistently, RT-qPCR indicated that the expression of circ_VANGL1 was enhanced in T24 and J82 cells relative to that in SV-HUC-1 cells (Figure 1b).

### 3.2 circ_VANGL1 knockdown suppressed cell viability, promoted cell apoptosis, and elevated doxorubicin sensitivity of bladder cancer cells

**Figure 2:**
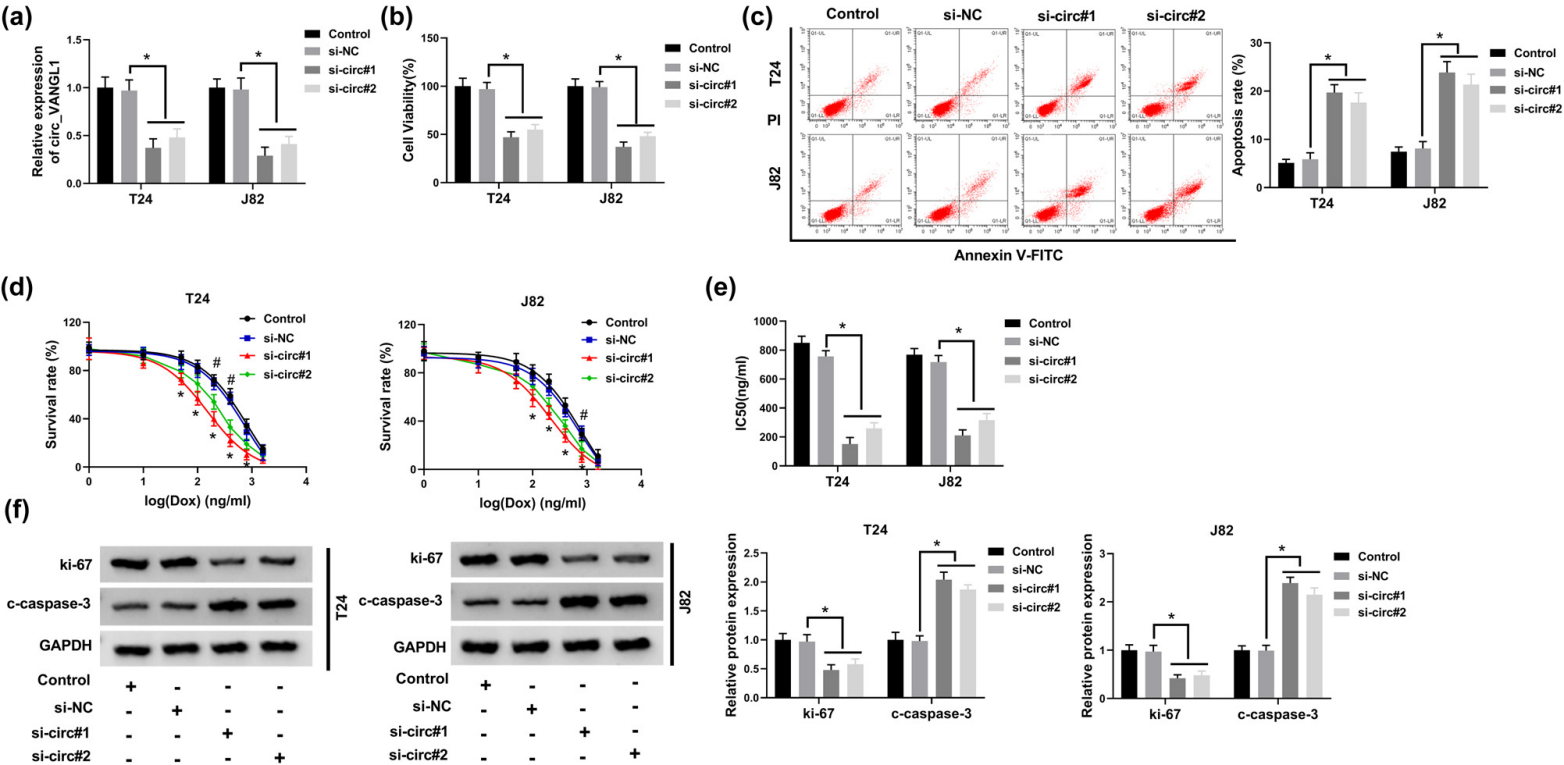
The effect of circ_VANGL1 knockdown on cell viability, apoptosis, and doxorubicin resistance. (a–f) T24 and J82 cells were transfected with si-NC, si-circ#1, or si-circ#2, respectively. (a) The expression level of circ_VANGL1 in transfected T24 and J82 cells. (b) Cell viability was measured by CCK-8 assay. (c) Flow cytometry analysis was performed to assess cell apoptosis rate. (d) Different concentrations of doxorubicin were incubated with the transfected cells, and then cell apoptosis rate was determined by flow cytometry analysis. (e) IC_50_ values of the growth of T24 and J82 cells treated with doxorubicin. (f) The protein levels of c-caspase-3 and ki-67 in T24 and J82 cells. *P < 0.05, ^#^ *P* < 0.05.

To investigate the effect of circ_VANGL1 on the progression of bladder cancer, T24 and J82 cells were transfected with si-NC or si-circ (si-circ#1 or si-circ#2). RT-qPCR assay revealed that the level of circ_VANGL1 was suppressed more than half in cells transfected with si-circ#1 or si-circ#2, especially in cells with si-circ#1 transfection (Figure 2a). Next, CCK-8 assay and flow cytometry were performed. The results suggested that T24 and J82 cells transfected with si-circ#1 or si-circ#2 exhibited lower cell viability (Figure 2b) and increased cell apoptosis rate in bladder cancer cells (Figure 2c). Meanwhile, CCK-8 assay was performed to detect the survival rate and IC_50_ values of T24 and J82 cells with doxorubicin treatment and si-circ#1 or si-circ#2 transfection. The results revealed that circ_VANGL1 knockdown decreased cell survival rate and IC_50_ value of doxorubicin in the T24 and J82 cells, which was shown by a statistical difference between the si-NC group and si-circ#1 or si-circ#2 group (Figure 2d and e). Moreover, we also detected the levels of apoptosis-related protein c-caspase-3 and proliferation-related protein ki-67 in T24 and J82 cells. Our data suggested that knockdown of circ_VANGL1 decreased ki-67 protein level, but increased c-caspase-3 protein level in both T24 and J82 cells (Figure 2f). These data demonstrated that circ_VANGL1 could enhance cell viability and inhibit cell apoptosis and doxorubicin sensitivity in bladder cancer cells.

### 3.3 circ_VANGL1 acted as a molecular sponge of miR-145-5p

**Figure 3:**
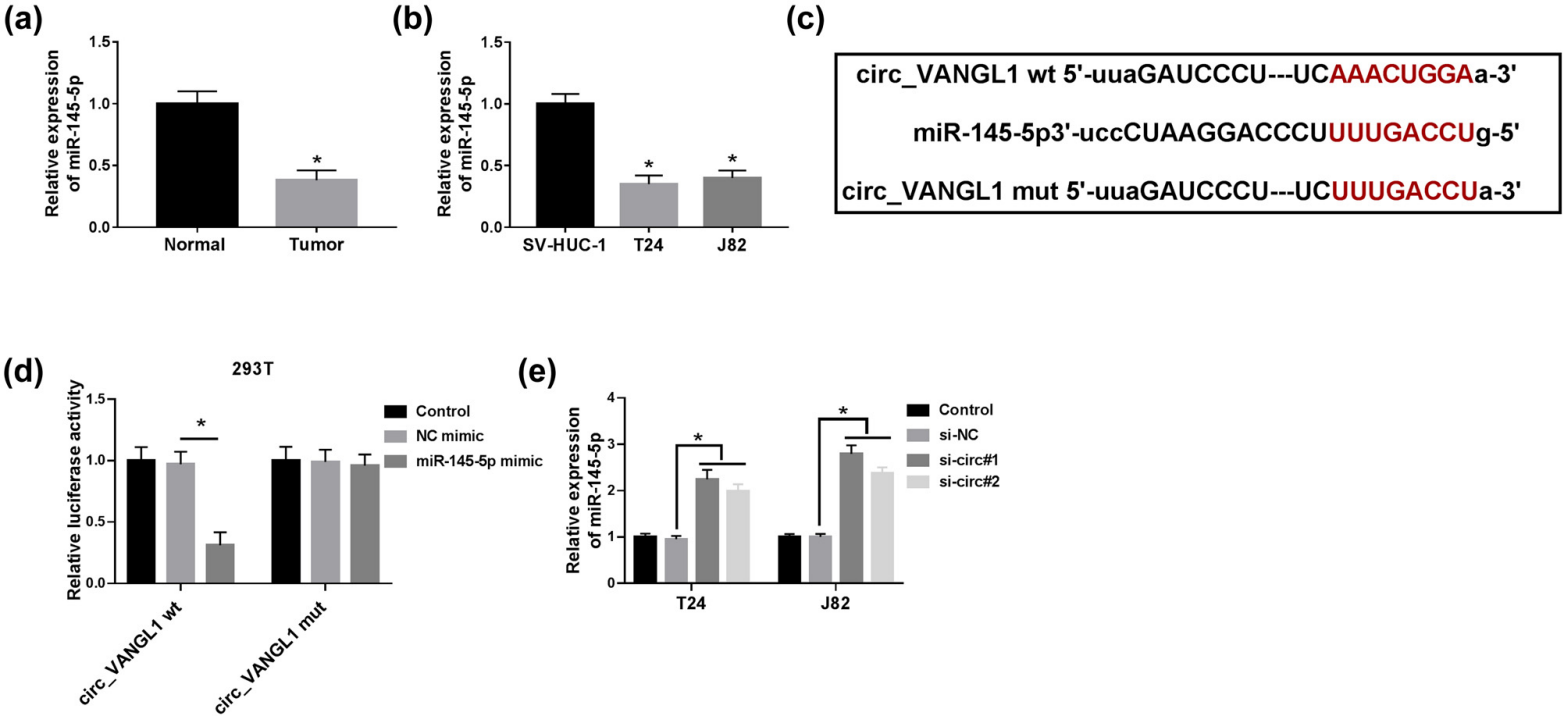
miR-145-5p was a target of circ_VANGL1. (a and b) RT-qPCR was used to detect the level of miR-145-5p in bladder cancer tissues (*n* = 36) and cells. (c) Schematic of the putative or mutant binding sites of miR-145-5p in circ_VANGL1 that predicted by starBase v2.0 tool. (d) The luciferase activity in 293T cells co-transfected with miR-145-5p mimic or NC-mimic and circ_VANGL1 wt or circ_VANGL1 mut. (e) T24 and J82 cells were transfected with si-NC, si-circ#1, or si-circ#2, respectively. And the expression level of miR-145-5p was examined by RT-qPCR. *P < 0.05.

**Figure S1:**
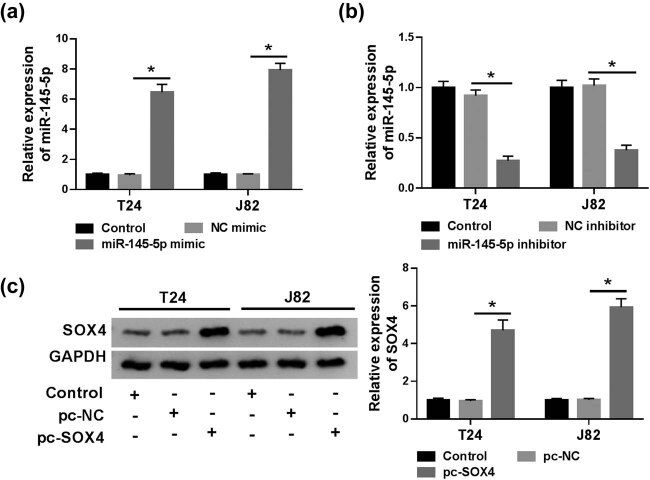
Transfection efficiency of miR-145-5p mimic, miR-145-5p inhibitor, and pc-SOX4 in T24 and J82 cells. (a) T24 and J82 cells was transfected with NC mimic or miR-145-5p mimic, respectively. And the expression of miR-145-5p was detected by RT-qPCR. (b) The expression of miR-145-5p in cells transfected with NC inhibitor or miR-145-5p inhibitor were evaluated by RT-qPCR. (c) T24 and J82 cells was transfected with pc-NC or pc-SOX4, respectively. And the protein level of SOX4 was detected by western blot. *P < 0.05.

To further explore the mechanism underlying circ_VANGL1 in bladder cancer progression, the directly interacted miRNAs of circ_VANGL1 were predicted by StarBase v2.0. We found that miR-145-5p might be a direct target of circ_VANGL1. RT-qPCR assay indicated that miR-145-5p was prominently downregulated in bladder cancer tumor tissues (*n* = 36) and cells, compared with matched normal tissues (*n* = 36) and cells (Figure 3a and b). To confirm the target relationship between circ_VANGL1 and miR-145-5p, circ_VANGL1 sequences containing wild-(circ_VANGL1 wt) or mutant-type (circ_VANGL1 mut) miR-145-5p binding sites were inserted into the luciferase reporter vector (Figure 3c). Transfection efficiencies of miR-145-5p mimic and inhibitor in T24 and J82 cells were detected. As shown in Figure S1a and b, miR-145-5p expression was enhanced six times by miR-145-5p mimic, but was inhibited in half by miR-145-5p inhibitor. Dual-luciferase reporter assay indicated that transfection of miR-145-5p suppressed the luciferase activity of circ_VANGL1 wt group compared with cells with NC-mimic transfection, while it had little effect on the luciferase activity of circ_VANGL1 mut group (Figure 3d). Moreover, miR-145-5p expression was significantly increased in T24 and J82 cells with si-circ#1 or si-circ#2 transfection (Figure 3e). These results indicated circ_VANGL1 as a sponge of miR-145-5p.

### 3.4 circ_VANGL1 targeted miR-145-5p to elevate cell viability, repress cell apoptosis, and attenuate doxorubicin sensitivity in bladder cancer cells

**Figure 4:**
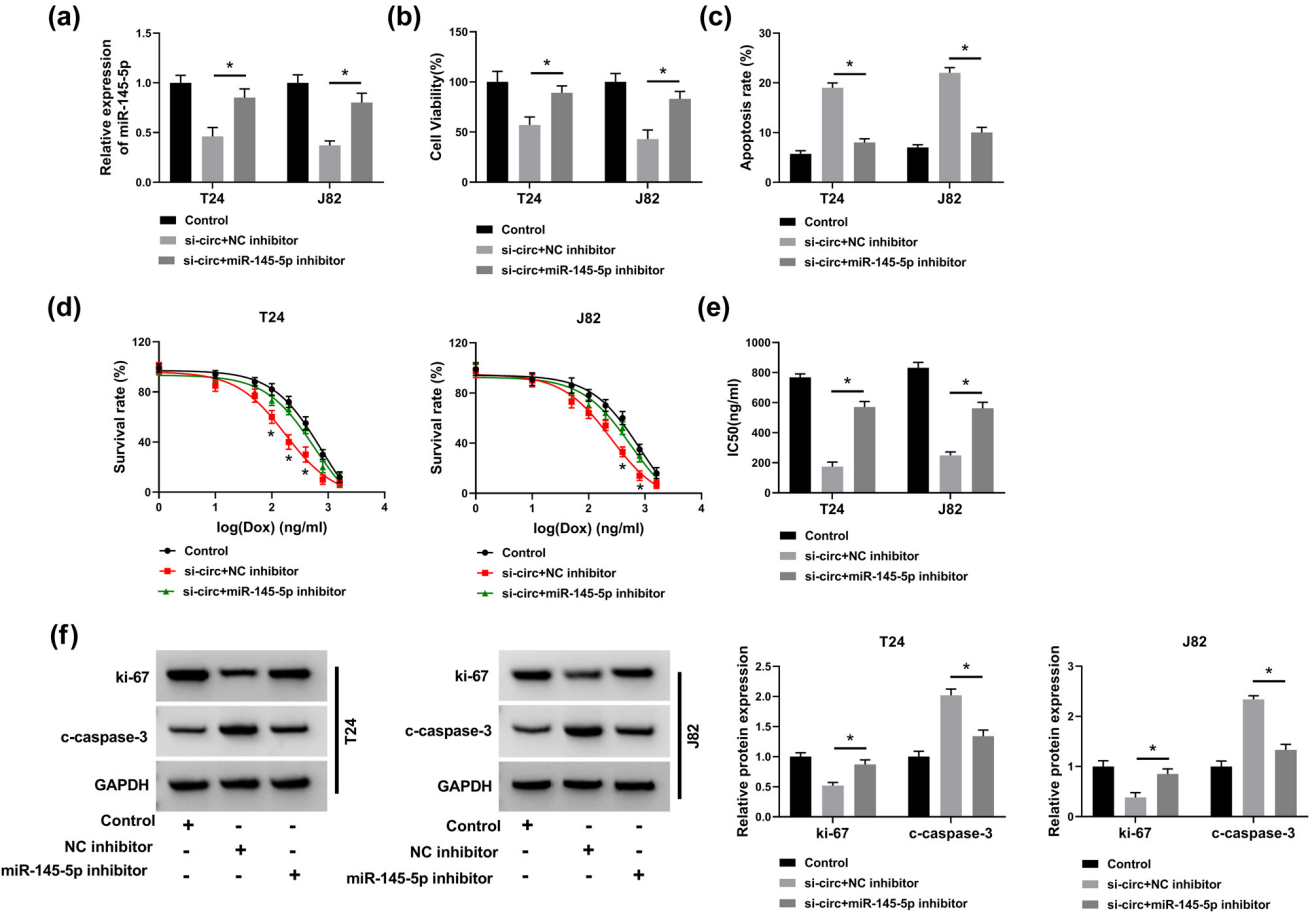
circ_VANGL1 targeted miR-145-5p to elevate cell viability, repress cell apoptosis, and reduce doxorubicin sensitivity in bladder cancer cells. (a–f) T24 and J82 cells were transfected with si-circ + NC inhibitor or si-circ + miR-145-5p inhibitor, respectively. (a) The level of miR-145-5p was determined by RT-qPCR. (b) CCK8 assay was performed to detect cell viability. (c) Cell apoptosis rate was detected by flow cytometry analysis. (d) T24 and J82 cells treated with different concentrations of doxorubicin, and cell survival rate was determined by flow cytometry analysis. (e) The IC_50_ values for the growth of the transfected T24 and J82 cells treated with doxorubicin. (f) Western blot was used to detect the protein levels of c-caspase-3 and ki-67. *P < 0.05.

Next, we investigated the regulatory mechanism of circ_VANGL1 and miR-145-5p in bladder cancer. As shown in Figure 4a, the decrease of miR-145-5p level induced by circ_VANGL1 knockdown was partially reversed by miR-145-5p inhibitor. CCK-8 results suggested that the inhibition effect of circ_VANGL1 knockdown on cell viability was attenuated by miR-145-5p inhibitor (Figure 4b). Moreover, circ_VANGL1 deletion-induced cell apoptosis was blocked by miR-145-5p downregulation (Figure 4c). Our data indicated that circ_VANGL1 depletion induced a significant decrease in the cell survival rate and IC_50_ of doxorubicin in T24 and J82 cells treated with doxorubicin, which were partially abolished by miR-145-5p inhibitor (Figure 4d and e). Western blot results revealed that miR-145-5p inhibitor reversed the suppression effect on ki-67 expression and the promotion effect on c-caspase-3 expression that were induced by circ_VANGL1 knockdown (Figure 4f). Overall, circ_VANGL1 regulated cell viability, apoptosis, and the doxorubicin sensitivity in T24 and J82 cells by sponging miR-145-5p.

### 3.5 SOX4 was directly targeted and inhibited by miR-145-5p

**Figure 5:**
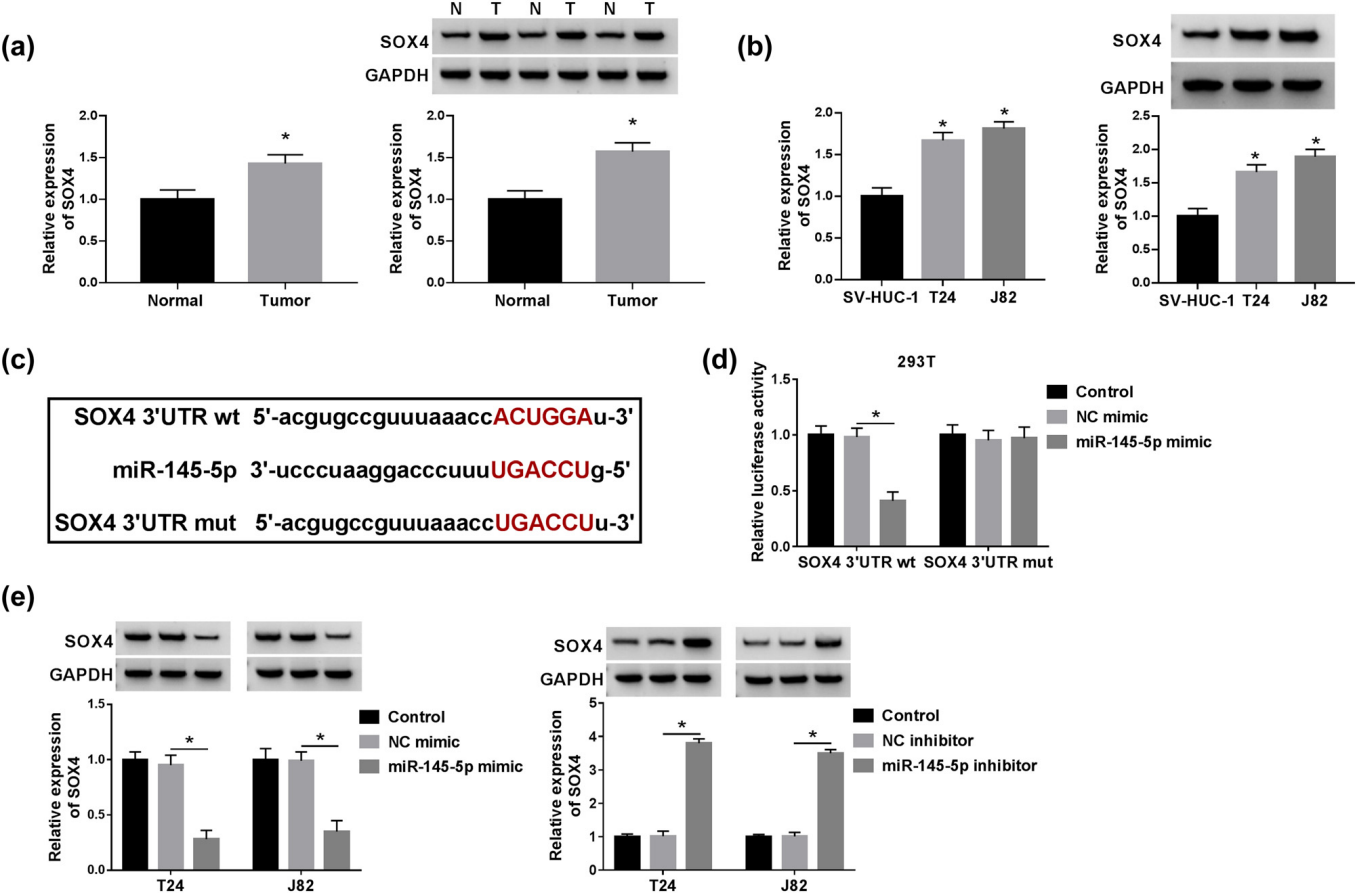
SOX4 was directly targeted and inhibited by miR-145-5p. (a) The mRNA and protein levels of SOX4 in bladder cancer tissues (*n* = 36) and normal tissues (*n* = 36) were detected by RT-qPCR and western blot. (b) The mRNA and protein levels of SOX4 in SV-HUC-1, T24, and J82 cells were measured by RT-qPCR. (c) The putative or mutant miR-145-5p binding sites in the 3′UTR of SOX4 were predicted by Targetscan tool. (d) 293T cells were co-transfected with NC-mimic or miR-145-5p mimic and SOX4 3′UTR wt or SOX4 3′UTR mut, and the luciferase activity was detected. (e) T24 and J82 cells were transfected with NC-mimic, miR-145-5p mimic, NC inhibitor, or miR-145-5p inhibitor, respectively. And the expression level of SOX4 was examined by western blot. *P < 0.05.

**Figure S2:**
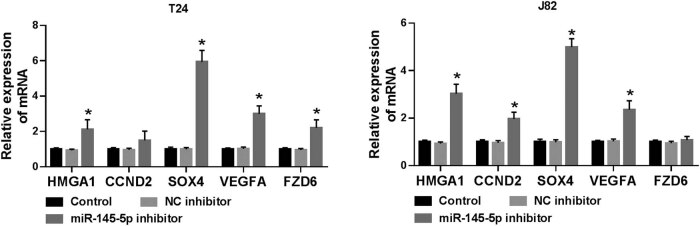
Effect of miR-145-5p inhibitor on the expression of its target mRNAs. T24 and J82 cells was transfected NC inhibitor or miR-145-5p inhibitor, respectively. And the mRNA levels of HMGA1, CCND2, SOX4, VEGFA and FZD6 were assessed by RT-qPCR. *P < 0.05.

To investigate the role of miR-145-5p in bladder cancer cell progression, the molecular targets of miR-145-5p were predicted by Targetscan software. As shown in Figure S2, HMGA1, CCND2, SOX4, VEGFA, and FZD6 expression were altered in T24 and J82 cells. And SOX4 expression was most significantly upregulated in cells transfected with miR-145-5p inhibitor. Besides, the expression of SOX4 in bladder cancer cells was evaluated. As described in Figure 5a and b, SOX4 expression was upregulated in tumor tissues (*n* = 36) and cells compared with that in normal tissues (*n* = 36) and cells. To verify the correlation between miR-145-5p and SOX4, Luciferase reporter plasmid vector containing the 3′UTR of wild-type SOX4 (SOX4 3′UTR wt) and mutant-type binding sites of miR-145-5p SOX4 (SOX4 3′UTR mut) was constructed to carry out the dual-luciferase reporter assay (Figure 5c). The results manifested that luciferase activity was diminished by SOX4 3′UTR wt and miR-145-5p, but not SOX4 3′UTR mut and miR-145-5p (Figure 5d). Moreover, our data showed that the protein level of SOX4 was inhibited by miR-145-5p mimic and elevated by miR-145-5p inhibitor (Figure 5e). In summary, miR-145-5p directly targeted to SOX4.

### 3.6 SOX4 mediated the effect of miR-145-5p on cell progression in bladder cancer cells

**Figure 6:**
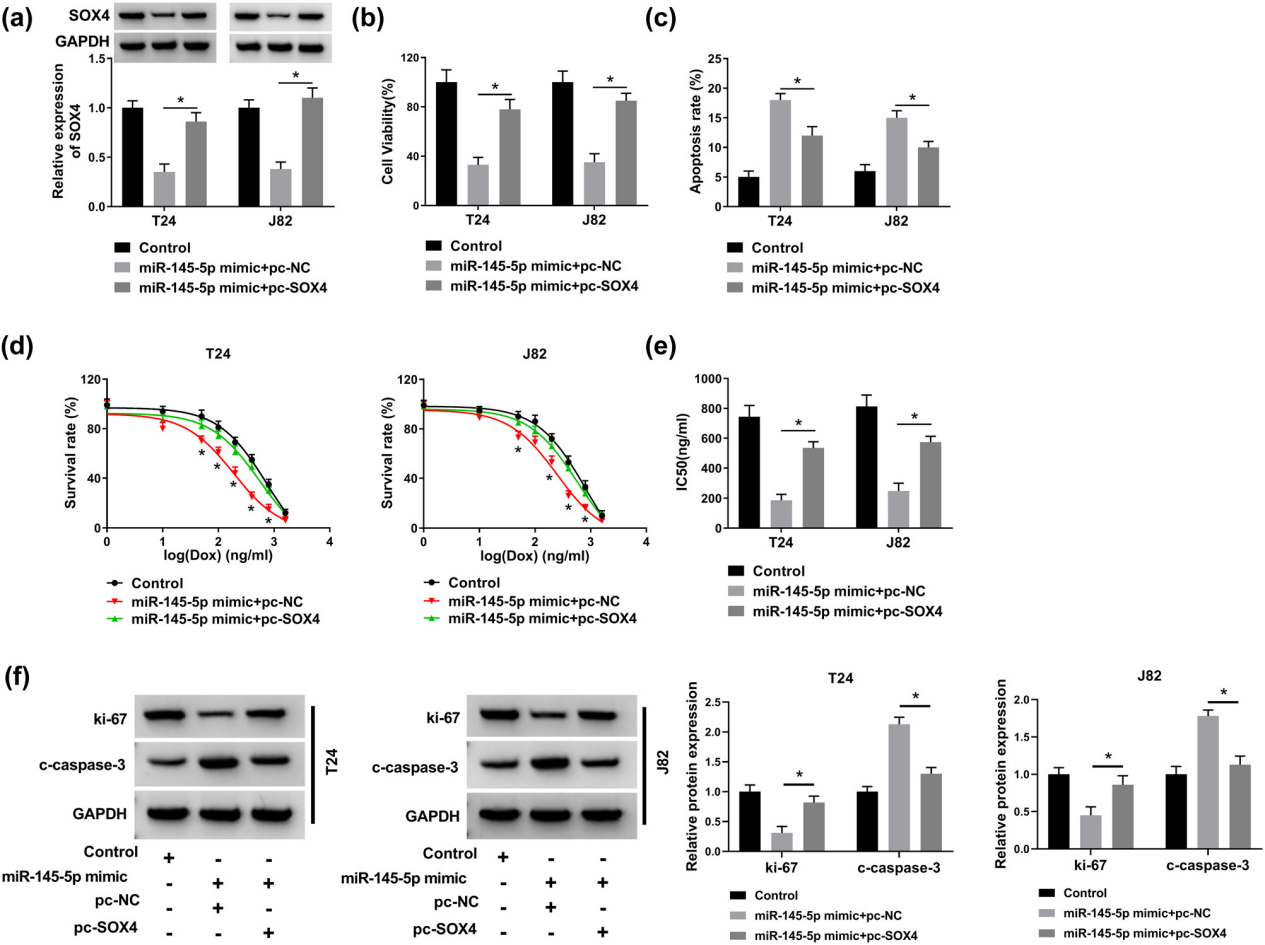
Overexpression of SOX4 weakened the effect of miR-145-5p on bladder cancer cells. (a–f) T24 and J82 cells were transfected with miR-145-5p mimic + pc-NC or miR-145-5p mimic + pc-SOX2, respectively. (a) The protein level of SOX4 was determined western blot after co-transfection with miR-145-5p mimic and pc-NC or pc-SOX4. (b) CCK8 assay was performed to detect cell viability. (c) Cell apoptosis rate was detected by flow cytometry analysis. (d) T24 and J82 cells were incubated with different concentrations of doxorubicin and cell survival rate was determined by flow cytometry analysis. (e) The IC_50_ values of cells treated with doxorubicin. (f) The protein levels of c-caspase-3 and ki-67 in the transfected cells were determined by western blot. *P < 0.05.

To explore the role of SOX4 on miR-145-5p-mediated cell progression, co-transfection experiment of SOX4 and miR-145-5p was performed. First, transfection efficiency of pc-SOX4 was examined. The protein level of SOX4 was significantly improved by SOX4 overexpressed plasmid (Figure S1c). T24 and J82 cells were co-transfected with miR-145-5p mimic and pc-SOX4 or pc-NC and then the expression level of SOX4 was detected by western blot. The results suggested that overexpression of SOX4 partly reversed the suppression effect of miR-145-5p mimic on the protein level of SOX4 (Figure 6a). Besides, miR-145-5p suppressed cell viability and promoted cell apoptosis, whereas this effect was reversed by upregulation of SOX4 (Figure 6b and c). Meanwhile, cell survival rate and IC_50_ of doxorubicin were also measured in the transfected cells. It was observed that cell survival rate and IC_50_ of doxorubicin were significantly reduced by miR-145-5p mimic, but the effect was abated by co-transfection of SOX4 overexpression plasmid (Figure 6d and e). Western blot assay indicated that overexpression of SOX4 could partially reverse the downregulation of ki-67 expression and upregulation of c-caspase-3 expression induced by miR-145-5p mimic (Figure 6f). All data suggested that miR-145-5p regulated cell progression by affecting SOX4 in bladder cancer cells.

### 3.7 circ_VANGL1 knockdown inhibited tumor growth and enhanced doxorubicin sensitivity *in vivo*

**Figure 7:**
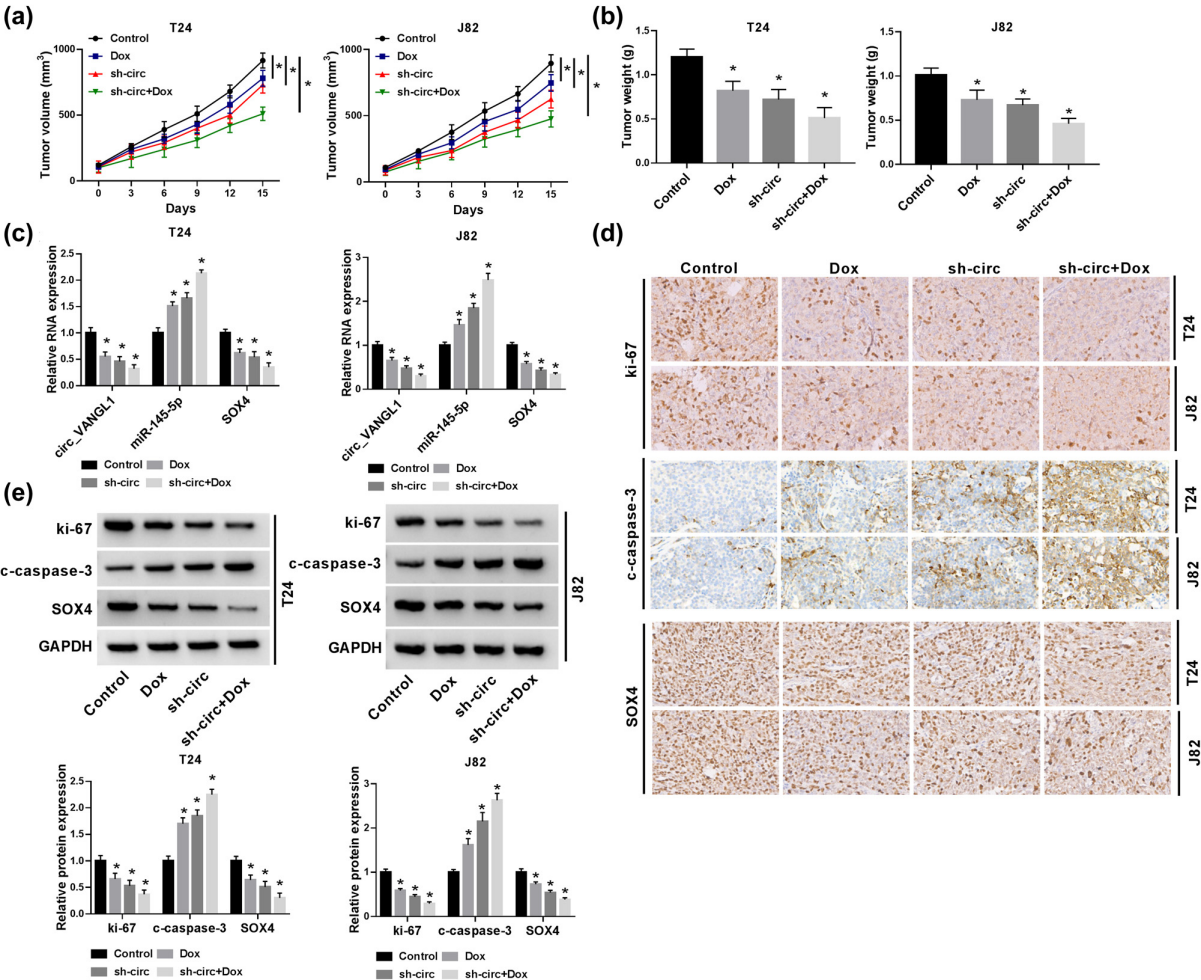
circ_VANGL1 knockdown inhibited tumor growth and enhanced doxorubicin sensitivity *in vivo*. T24 and J82 cells stably transfected with sh-circ or T24 and J82 cells were subcutaneously injected into the forelimb axils of nude mice, followed by the injection of doxorubicin (3 mg/kg) every 3 days. Fifteen days upon injection, the mice were killed to remove the tumor tissues. (a) Tumor volume was detected every 3 days for 15 days. (b) Tumor weight was measured. (c) The expressions of circ_VANGL1, miR-145-5p, and SOX4 in xenograft tissues were determined by RT-qPCR. (d) Proliferation in xenograft tumors was assessed by Ki67 Immunohistochemistry. (e) The protein levels of SOX4, c-casepase-3, and ki-67 were assessed by western blot. *P < 0.05.

Next, we further explored the effect of circ_VANGL1 in bladder cancer *in vivo* using xenograft mice model. In contrast with control group, the tumor volume and weight in mice with doxorubicin treatment were declined and further inhibited by circ_VANGL1 knockdown, as well as co-treatment of doxorubicin and sh-circ (Figure 7a and b). Besides, the expression of circ_VANGL1 and SOX4 was downregulated, while miR-145-5p was upregulated in xenograft tumor tissues with doxorubicin treatment, circ_VANGL1 knockdown, and co-treatment of doxorubicin and circ_VANGL1 knockdown (Figure 7c). Using ki-67 as a marker for proliferation, immunohistochemistry showed a significant reduction in proliferation in the mice with doxorubicin treatment, circ_VANGL1 knockdown, and co-treatment of doxorubicin and circ_VANGL1 knockdown (Figure 7d). Besides, we found that the protein levels of ki-67 and SOX4 were decreased and c-caspase-3 was increased in doxorubicin, circ_VANGL1 deletion, and co-treatment of doxorubicin and circ_VANGL1 deletion group (Figure 7e). These results illustrated that circ_VANGL1 knockdown could suppress tumor growth and increase the doxorubicin sensitivity *in vivo*.

## 4 Discussion

In the present study, we focused on the role of circ_VANGL1 and its effects on cell growth, apoptosis, and the doxorubicin sensitivity in bladder cancer *in vitro* and *in vivo* through molecular and functional experiments and revealed that circ_VANGL1 served as a sponger of miR-145-5p to regulate SOX4 expression. Our data suggested that circ_VANGL1 might be a potential therapeutic target for the treatment of bladder cancer.

Previous studies reported that circ_VANGL1 was increased in non-small cell lung cancer to affect tumor progression [14] and upregulated in bladder cancer [15,16]. Consistently, a significant increase of circ_VANGL1 level was observed in bladder cancer tissues and cell lines, indicating the tumor promotion role in bladder cancer. Herein, loss-function-assays showed that circ_VANGL1 deletion repressed cell viability, induced cell apoptosis, and decreased the resistance to doxorubicin in bladder cancer cells *in vitro*. Meanwhile, these promotion effects were also supported by *in vivo* experiments.

StarBase v2.0 showed that miR-145-5p was a target gene of circ_VANGL1, which was confirmed by dual-luciferase reporter assay. Previous studies reported that miR-145-5p was prostate carcinoma [17], breast cancer [18], and bladder cancer [19]. Consistent with the previous research, we found that miR-145-5p was lowly expressed in bladder cancer tissues and cells. Previous evidence proved that circRNAs mainly served as sponge of miRNAs and caused a loss function of miRNA accompanied with upregulated expression of their endogenous targets [5,20]. For instance, CDR1as was involved in hepatocellular carcinoma and colorectal cancer by regulating miR-7 [21,22]. Another study suggested that cir-HIPK3 derived from exon 2 of the HIPK3 was proved to function as a sponge for miR-124 [23]. Moreover, hsa_circ_0007843 elevated cell metastasis of SW480 cells by targeting miR-518c-5p and hsa_circ_RNA_0011780 sponged miR-544a to suppress cell growth, migration, and invasion in non-small cell lung cancer [24,25]. In the present study, we observed that miR-145-5p inhibitor could partially reverse the effects of circ_VANGL1 deletion on cell viability, apoptosis, and the resistance to doxorubicin, suggesting a regulatory network of circ_VANGL1/miR-145-5p in bladder cancer progression.

SOX4, a sox transcription factor, has been demonstrated to play an oncogenic role and was related to tumor progression and development [26]. Multiple researches reported that SOX4 could be regulated by lots of miRNAs in human cancers [27]. For example, miR-140-5p directly targeted SOX4 to participate in tumorigenesis in malignant melanoma [28]. Furthermore, miR-195 repressed cell metastasis and epithelial-mesenchymal transition by regulating the expression of SOX4 in endometrial carcinoma [29]. An earlier study also showed that miR-191-5p increased the doxorubicin sensitivity by targeting SOX4 in breast cancer cells [30]. In our research, the relationship between miR-145-5p and SOX4 was predicted by Targetscan and was verified by dual-luciferase reporter assay. Moreover, our results revealed that SOX4 expression was enhanced in bladder cancer tissues and cells. Besides, miR-145-5p could negatively regulate the expression of SOX4. Rescue experiments demonstrated that the inhibitory effects on cell viability, resistance to doxorubicin, and promotion effects on cell apoptosis were partially blocked by SOX4 overexpression. Thus, we concluded that miR-145-5p exerts it function through the regulation of SOX4.

**Figure 8:**
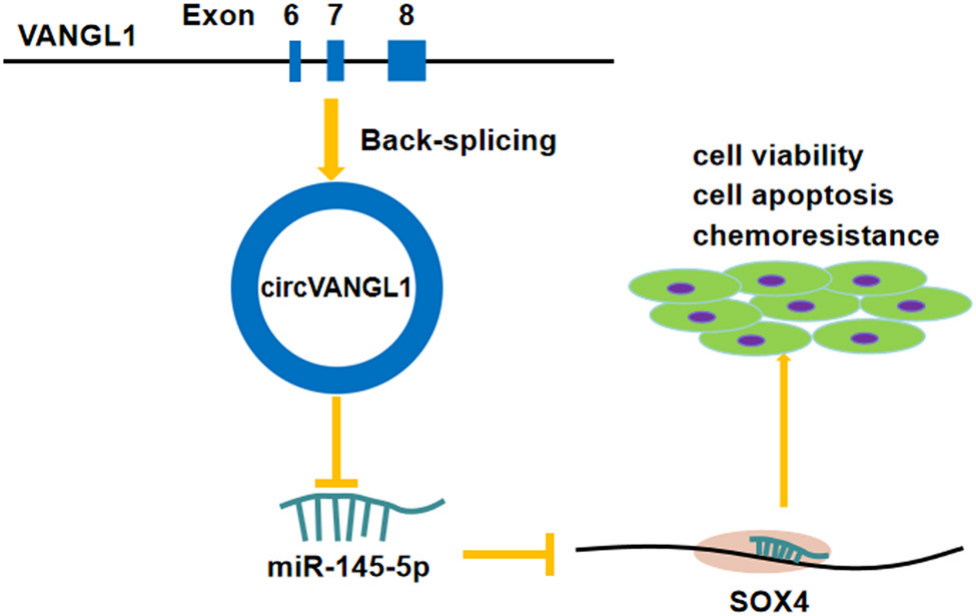
Schematic diagram indicates the regulation of circ_VANGL1/miR-145-5p/SOX4 axis in bladder cancer cells.

In conclusion, we found that the expressions of circ_VANGL1 and SOX4 were enhanced, while miR-145-5p was inhibited in bladder cancer tissues and cells. Moreover, circ_VANGL1 promoted cell viability, suppressed cell apoptosis, and increased the doxorubicin sensitivity *in vitro* and *in vivo*. Furthermore, we proposed a novel circ_VANGL1/miR-145-5p/SOX4 signaling regulatory network in bladder cancer (Figure 8), which might provide a potential biomarker and therapeutic target for bladder cancer.
